# Comparison of the night-time effectiveness in achieving glycemic targets in adults with type 1 diabetes of three advanced hybryd closed-loop systems

**DOI:** 10.1007/s00592-024-02397-9

**Published:** 2024-11-22

**Authors:** Nicolò Diego Borella, Antonio Ferramosca, Giona Castagna, Silvia Ippolito, Sara Ceresoli, Antonio Taverna, Beatrice Sonzogni, Roberto Trevisan, Giuseppe Lepore

**Affiliations:** 1 Unit of Endocrine Diseases and Diabetology, Department of Medicine, ASST Papa Giovanni XXIII, Piazza OMS, 1, 24127 Bergamo, Italy; 2 Control Systems and Automation Laboratory, Department of Management, Information and Production Engineering, University of Bergamo, Bergamo, Italy; 3 Department of Medicine and Surgery, University of Milano Bicocca, Milan, Italy

## Abstract

**Context:**

Advanced hybrid closed loop (AHCL) systems currently represent the most advanced modality of insulin therapy.

**Aim:**

To compare the night-time (from 00 to 07 a.m.) effectiveness in achieving recommended glycemic targets of three different AHCL systems in adults with type 1 diabetes (T1D).

**Methods:**

We retrospectively evaluated 55 adults with T1D (mean age 41 ± 16 years, male 40%, diabetes duration 19.4 ± 11.4 years, BMI 24.1 ± 4.1 kg/m^2^) with similar glycemic control (GMI 7.0–7.4%). Twenty-two participants were using the Minimed 780G system, 18 the Tandem t:slim X2 with Control-IQ system and 15 the DBLG1 system.

Continuous glucose monitoring derived metrics and insulin requirement of 14 consecutive nights were analyzed.

**Results:**

All three groups achieved the recommended mean TIR > 70%, mean TBR < 4%, and mean CV < 36% with a similar insulin requirement (Minimed 780G system: TIR 73.9 ± 11.2%, TBR 0.9 ± 1.2%, CV 29 ± 6.7%; Tandem t:slim X2 with Control-IQ system: TIR 74.1 ± 11.1%, TBR 1.1 ± 1.0%, CV 34.5 ± 6.6%; DBLG1 System TIR 71.7 ± 11.3%, TBR 1.4 ± 3.7%, CV 32.4 ± 7.1%). Tight TIR% (70–140 mg/dl) was significantly higher (p < 0.01) in the Tandem t:slim X2 with Control-IQ group (51.5 ± 9.8%) when compared to Minimed 780G group (42.1 ± 13.7%) and DBLG1 System (40.1 ± 10.5%). In all three groups the insulin infusion similarly decreased from midnight to 05.00 am and then increased.

**Conclusions:**

All the three AHCL systems achieved the recommended TIR, TBR and CV without difference in insulin requirement. The Tandem Control-IQ system obtained a higher tight TIR.

## Introduction

Advanced hybrid closed-loop (AHCL) systems represent the state of art of insulin therapy in people with type 1 diabetes (T1D). They combine three components: a continuous subcutaneous insulin pump, a sensor for continuous glucose monitoring (CGM), and a control algorithm that constantly analyzes the sensor-measured interstitial glucose and adjusts the rate of basal insulin accordingly. Manual meal boluses are still required.

Currently, the Italian National Health System allows funded access to several AHCL systems approved for adults: the MinimedTM 780G system (Minimed Medtronic, Northridge, California, USA), the Tandem t:slim X2 Control IQTM system (Control-IQ technology, Tandem Inc., San Diego, CA, USA) the Diabeloop Generation 1 (DBLG1® system, Grenoble, France), and the CamAPS FX app (CamDiab, Cambridge, UK).

These systems have demonstrated in several RCTs [1–3] and real-world studies [4–7] to better achieve and maintain glycemic targets in people with type 1 diabetes compared to multiple dose insulin (MDI) therapy, conventional continuous subcutaneous insulin infusion (CSII) or to sensor-augmented pump (SAP) with predictive low-glucose suspend systems. Few, most pediatric, studies compared the real-life efficacy of different AHCL systems [8–10]. However, in everyday real life, many confounders can make it difficult to compare different AHCL systems, such as lifestyle during the day, the precision of the insulin bolus at meals, the degree of physical activity and, last but not least, differences in the overall degree of glycemic control between diabetic subjects.

Precisely for this reason we decided to compare the effectiveness of AHCL systems with different control algorithms in a group of patients with similar glycemic control and only during the night-time hours to minimize confounding effects.

Therefore, the aim of this study was to compare the effectiveness of three different AHCL systems in adults with T1DM with an almost good glycemic control in achieving the recommended glycemic targets at night-time (00:00 to 07:00).

## Materials and methods

We retrospectively evaluated 55 adults with type 1 diabetes mellitus (T1DM) followed by the Unit of Endocrine Diseases and Diabetology, ASST Papa Giovanni XXIII (Bergamo, Italy), who were already using an AHCL system for at least 6 months.

Inclusion criteria was a similar daily glycemic control with a Glucose Management Index (GMI) between 7.0% and 7.4% in the last 3 months before the night-time comparison. Furthermore, all subjects had dinner before 9 pm and did not start breakfast before 7am during the 14-day study period.

Twenty-two were using the Medtronic MinimedTM 780G system composed by an insulin pump containing a Predictive Integrative Derivative (PID) algorithm (SmartGuard algorithm) and by Guardian G4 sensor; 18 were using a system combining an insulin pump Tandem t:slim X2 integrated with a Model Predictive of Control (MPC) algorithm and a Dexcom G6 continuous glucose monitoring sensor (Dexcom Inc., San Diego, CA, USA), and 15 were using a system combining an insulin pump Accu-Chek Insight (Roche Diabetes CARE, Basel, Switzerland), a Dexcom G6 continuous glucose monitoring device and a Model Predictive Control algorithm (Diabeloop Generation 1) contained in a controller handset.

Demographic, anamnestic, and clinical data of the participants [i.e., age, gender, weight, height, body mass index (BMI), duration of diabetes], were obtained from the electronic medical record.

Data regarding percentage time spent in hypoglycemic (TBR < 54 and TBR 54–69 mg/dl), euglycemic (TIR 70–180 mg/dl and tight TIR 70–140 mg/dl), and hyperglycemic (TAR 181–250 and TAR > 250 mg/dl) ranges, CGM-measured mean glucose concentration, glucose management indicator (GMI), coefficient of variation (CV) of CGM-measured glucose concentrations, percentage of sensor use, and insulin infusion of 14 consecutive days were downloaded from the platforms Carelink,, Glooko-Diasend and Yourloops.

The participants were categorized in three groups according to the AHCL system they were using (Minimed 780G group, Control-IQ group and DBLG1 group).

### Outcomes

Main outcomes were the differences between the three groups in CGM metrics and in insulin infusion in the night-time period from 00:00 to 07:00.

### Statistical analysis

Data are shown as mean ± SD.

All p values are two sided. A p value < 0.05 was considered significant.

All variables were tested to evaluate whether they were normally distributed using the Kolmogorov–Smirnov test. Depending on the distribution, comparison tests were performed using analysis of variance ANOVA (normally distributed data) and Kruskal–Wallis (non-normally distributed data).

To analyze insulin dosage changes over night, we employed a panel data regression model to examine temporal variations. The first time-interval (00:00 to 01:00) served as the reference parameter. We calculated predicted margins to estimate the changes over time and employed a parameter test to assess the influence of the time of day on insulin dosage.

To assess nocturnal variations in insulin dosage across the three AHCL systems, we compared the administered insulin doses within each hourly interval (00:00 to 01:00, 01:00 to 02:00, …) using the Kruskal–Wallis test.

Analyses were carried out with Stata.

The study was performed in accordance with the Helsinki Declaration of 1964 and its later amendments and agreed with national regulations. It was approved by the local ethical committee and written informed consent to use the clinical and biochemical data was obtained from each participant.

## Results

Baseline demographic and clinical characteristics of the three groups are shown in Table 1.

**Table 1:** Baseline demographic, clinical characteristics and CGM metrics (during the 14-day study period) of participants

	All participants (n = 55)	Minimed780 G group (n = 22)	Control-IQ group (n = 18)	DBLG1 group (n = 15)	*P*
Age (years)	41.2 ± 15.6	47.6 ± 14.9	34.6 ± 12.4	39.8 ± 16.7	0.03*
Sex (female), n(%)	33 (60)	14 (63,6)	11 (61,1)	8 (53,3)	0.82
BMI (kg/m^2^)	24.1 ± 4.1	23.8 ± 4	24 ± 3.7	24.7 ± 5	0.90
Age of onset T1D (years)	21.8 ± 14	23.3 ± 13.6	17.7 ± 11.5	24.5 ± 5	0.31
T1D duration (years)	19.4 ± 11.4	24.3 ± 13.2	16.9 ± 7.1	15.3 ± 10.5	0.03°
Insulin Daily dose (U/kg)	0.6 ± 0.3	0.6 ± 0.3	0.6 ± 0.2	0.6 ± 0.3	0.73
Glucose Management Indicator %	7.2 ± 0.1	7.2 ± 0.1	7.2 ± 0.2	7.2 ± 0.1	0.48
TIR % (70–180 mg/dl)	66.6 ± 5.1	66.2 ± 4.8	67.1 ± 5.6	66.7 ± 5.1	0.86
TAR % (181–250 mg/dl)	24.6 ± 3.9	25.5 ± 3.6	24.2 ± 4	23.8 ± 4	0.38
TAR % (> 250 mg/dl)	7.7 ± 2.9	7.4 ± 2.9	7.8 ± 3.3	8.1 ± 2.5	0.76
TBR % (69–54 mg/dl)	1 ± 0.8	0.8 ± 0.8	0.8 ± 0.9	1.1 ± 0.8	0.75
TBR % (< 54 mg/dl)	0,1 ± 0,4	0,1 ± 0,3	0,1 ± 0,2	0,3 ± 0,7	0.73
Sensor mean glucose (mg/dl)	162.9 ± 6.1	163.5 ± 5.3	161.8 ± 7.2	163.5 ± 6	0.64
Coefficient of variation %	33.1 ± 4.6	33.3 ± 4.7	34.8 ± 4.2	31.3 ± 4.3	0.10
Sensor use %	95.6 ± 4.9	93,3 ± 5.8	98.1 ± 1.49	96 ± 4.9	< 0.001*°
Auto mode (smart guard) %	95 ± 7.9	95.8 ± 4.9	91.4 ± 14.8	95.3 ± 6.9	0.28

Data are expressed as mean ± SD unless otherwise indicated

Statistically significant difference between Minimed 780G and Contro-IQ: *, between Minimed 780G and DBLG1: °

Although the subjects in the Diabeloop group and especially those in the Control-IQ group were younger than those in Minimed 780G group, the 3 groups were similar with regard to BMI, age at onset and duration of diabetes, and daily insulin requirement. As per the inclusion criterion, the CGM metrics were comparable in the 3 groups at the time of the 14 days of the study period.

The CGM metrics, the insulin requirement, and the percentage of sensor and of automatic mode use in the 14 days of the study did not differ between the three groups (Table 1).

### Night-time period

**Fig. 1:**
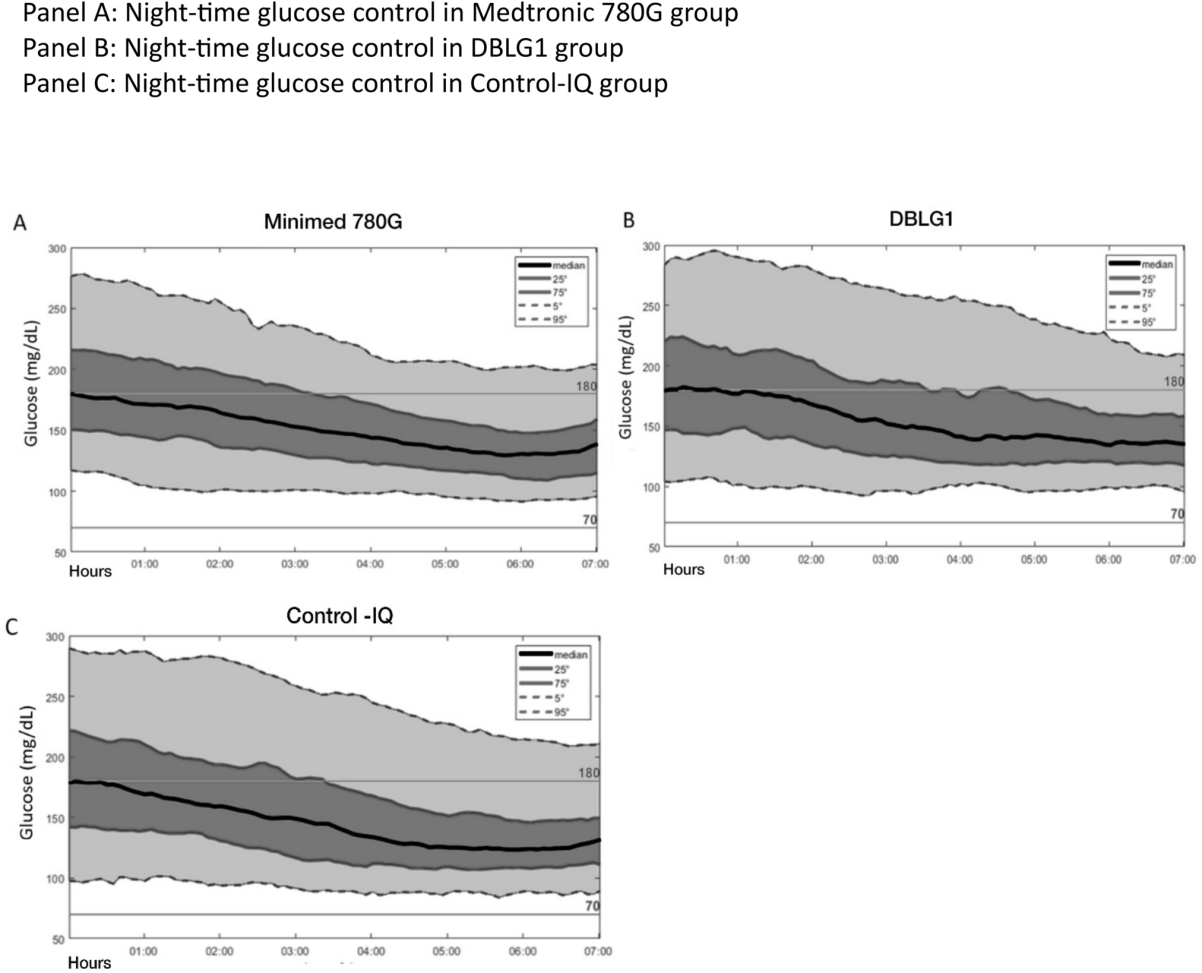
Night-time glucose trend in aHCL participants. **A**: Night-time glucose control in Medtronic 780G group. **B**: Night-time glucose control in DBLG1 group. **C**: Night-time glucose control in Control-IQ group

The trend of glucose was similar in the three groups with a progressive reduction from 00:00 to 06:00 (Fig. 1A, B and C).

The Control-IQ group spent a significantly (p = 0.007) higher percentage of time in tight TIR (51.5 ± 9.8%) compared to the Minimed 780G group (42.1 ± 13.7%) and the DBLG1 group (40.1 ± 10.5%) (Table 2).

**Table 2:** Night-time CGM metrics in all paticipants, in Minimed 780G group, in Control-IQ group and in DBLG1 group

	Overnight	0–1	1–2	2–3	3–4	4–5	5–6	6–7	P
*Tight TIR % (70–140 mg/dl)*									
Total	44.6 ± 12.5	25.0 ± 16.9	29.0 ± 16.0	38.5 ± 16.7	45.1 ± 17.3	53.7 ± 18.4	59.4 ± 20.4	61.8 ± 20.1	< 0.01
Minimed 780G	42.1 ± 13.7	21.3 ± 15.3	25.6 ± 13.5	34.1 ± 17.1	39.8 ± 18.8	51.1 ± 20.5	59.9 ± 21.3	62.3 ± 19.6	< 0.01
Control-IQ	51.5 ± 9.8	28.8 ± 16.3	33.0 ± 18.1	43.4 ± 15.2	51.7 ± 14.1	62.5 ± 11.7	69.6 ± 14.2	70.9 ± 17.1	< 0.01
DBLG1	40.1 ± 10.5	26–0 ± 19.4	29.0 ± 16.7	38.8 ± 17.1	45.1 ± 16.9	46.7 ± 18.5	46.3 ± 19.2	50.1 ± 19.3	< 0.01
P	0.01*^#^	0.35	0.34	0.21	0.09	0.03*#	< 0.01#	0.01#	
*TIR % (70–180 mg/dl)*									
Total	73.4 ± 11.1	53.0 ± 18.3	60.2 ± 18.9	68.9 ± 17.5	75.9 ± 14.0	82.1 ± 11.5	86.4 ± 11.3	87.2 ± 12.6	< 0.01
Minimed 780G	73.9 ± 11.2	51.7 ± 16.8	59.9 ± 19.3	68.4 ± 18.6	75.4 ± 13.8	83.6 ± 10.4	89.3 ± 9.2	88.6 ± 9.4	< 0.01
Control-IQ	74.1 ± 11.1	52.8 ± 19.9	61.0 ± 20.4	68.6 ± 17.3	76.7 ± 11.9	83.6 ± 10.3	87.3 ± 11.1	88.3 ± 9.7	< 0.01
DBLG1	71.7 ± 11.3	55.0 ± 19.5	59.7 ± 18.0	69.8 ± 17.5	75.6 ± 17.2	77.8 ± 13.8	81.1 ± 13.1	83.6 ± 18.6	< 0.01
P	0.82	0.87	0.98	0.97	0.95	0.25	0.08	0.42	
*TAR % 181–250 mg/dl)*									
Total	19.9 ± 8.3	32.2 ± 15.5	28.1 ± 13.1	24.0 ± 13.4	18.3 ± 10.2	13.4 ± 8.8	8.7 ± 8.4	7.7 ± 9.9	< 0.01
Minimed 780G	20.7 ± 9	37.2 ± 17.2	31.0 ± 13.9	25.4 ± 13.5	19.5 ± 10.0	14.2 ± 9.1	7.2 ± 7.4	6.6 ± 6.4	< 0.01
Control-IQ	18.5 ± 6.8	31.8 ± 13.0	27.2 ± 12.8	23.9 ± 12.9	17.5 ± 8.5	11.8 ± 7.5	7.9 ± 7.5	6.8 ± 8.1	< 0.01
DBLG1	20.4 ± 9.3	25.3 ± 13.8	25.0 ± 12.0	22.2 ± 14.5	17.4 ± 12.5	14.1 ± 10.1	12.0 ± 10.2	10.4 ± 15.0	< 0.01
P	0.74	0.07	0.38	0.80	0.77	0.69	0.21	0.91	
*TAR % (> 250 mg/dl)*									
Total	5.7 ± 5.1	11.5 ± 8.2	8.8 ± 8.6	5.9 ± 7.8	4.6 ± 7.3	3.4 ± 5.9	2.8 ± 5.0	2.8 ± 4.6	< 0.01
Minimed 780G	4.5 ± 4.8	10.2 ± 7.8	7.8 ± 8.6	5.2 ± 9.2	3.7 ± 8.1	1.3 ± 2.7	1.7 ± 3.4	2.0 ± 4.3	< 0.01
Control-IQ	6.3 ± 5.9	14.3 ± 9.1	10.8 ± 9.5	6.6 ± 7.1	4.8 ± 7.4	3.1 ± 5.8	2.4 ± 5.2	2.3 ± 3.5	< 0.01
DBLG1	6.5 ± 4.7	9.9 ± 7.2	8.0 ± 7.7	6.1 ± 6.6	5.9 ± 6.2	6.7 ± 8.0	5.0 ± 6.1	4.5 ± 5.8	0.43
P	0.30	0.20	0.35	0.42	0.30	0.03°#	0.08	0.91	
*TBR % (69–54 mg/dl)*									
Total	0.7 ± 1.2	0.6 ± 1.4	1.1 ± 2.6	0.7 ± 2.1	0.7 ± 1.5	0.8 ± 1.8	0.8 ± 1.7	0.6 ± 1.5	0.95
Minimed 780G	0.7 ± 0.9	0.6 ± 1.3	1.3 ± 2.4	0.6 ± 1.1	0.8 ± 1.7	0.8 ± 1.7	1.7 ± 3.4	0.4 ± 1.5	0.99
Control-IQ	0.8 ± 0.8	0.6 ± 1.2	0.6 ± 1.0	0.5 ± 0.8	0.8 ± 1.3	1.2 ± 2.5	1.3 ± 2	0.8 ± 1.8	0.78
DBLG1	0.8 ± 1.9	0.7 ± 1.9	1.2 ± 3.9	1.2 ± 3.7	0.5 ± 1.3	0.3 ± 0.5	0.7 ± 1.6	0.5 ± 1.2	0.95
P	0.56	0.21	0.66	0.89	0.63	0.84	0.30	0.47	
*TBR % (< 54 mg/dl)*									
Total	0.3 ± 1.0	0.5 ± 1.9	0.3 ± 0.9	0.3 ± 1.0	0.4 ± 1.7	0.4 ± 1.8	0.2 ± 1.0	0.1 ± 0.5	0.99
Minimed 780G	0.2 ± 0.4	0.3 ± 1.3	0.0 ± 0.1	0.2 ± 0.9	0.6 ± 2.0	0.0 ± 0.1	0.0 ± 0.0	0.0 ± 0.0	0.99
Control-IQ	0.3 ± 0.3	0.5 ± 1.3	0.4 ± 1	0.2 ± 0.4	0.2 ± 0.5	0.3 ± 0.7	0.2 ± 0.7	0.3 ± 0.7	0.99
DBLG1	0.6 ± 1.8	0.9 ± 3.0	0.5 ± 1.2	0.6 ± 1.6	0.6 ± 2.2	1.0 ± 3.4	0.7 ± 1.8	0.2 ± 0.7	0.99
P	0.75	0.42	0.67	0.92	0.99	0.78	0.74	0.49	
*Sensor mean glucose (mg/dl)*									
Total	155.4 ± 14.5	179.9 ± 22.7	171.1 ± 22.3	161.6 ± 21.9	153.0 ± 20.8	145.5 ± 18.6	139.5 ± 18.0	138.0 ± 19.0	< 0.01
Minimed 780G	155.3 ± 14.0	181.5 ± 17.2	172.5 ± 18.3	163.0 ± 22.3	153.6 ± 21.2	143.8 ± 15.5	136.5 ± 17.0	135.7 ± 18.9	< 0.01
Control-IQ	152.9 ± 15.4	181.0 ± 25.9	172.2 ± 25.2	161.6 ± 21.4	150.0 ± 20.6	139.8 ± 19.6	133.8 ± 16.1	132.5 ± 14.8	< 0.01
DBLG1	158.5 ± 14.4	176.2 ± 26.6	170.0 ± 25.3	159.4 ± 23.3	155.7 ± 21.2	154.8 ± 19.1	150.8 ± 17.5	147.9 ± 21.0	0.01
P	0.54	0.76	0.94	0.89	0.73	0.06	0.01°#	0.047#	
*Coefficient of variation %*									
Total	31.7 ± 7.1	29.1 ± 9.0	28.9 ± 8.5	29.0 ± 8.1	28.5 ± 8.6	27.3 ± 9.7	26.4 ± 11.4	25.2 ± 9.4	0.06
Minimed 780G	29.0 ± 6.7	26.9 ± 9.8	27.2 ± 8.7	25.7 ± 7.8	24.5 ± 6.7	22.4 ± 6.3	23.0 ± 8.1	24.0 ± 8.5	0.42
Control-IQ	34.5 ± 6.6	31.3 ± 8.0	31.1 ± 8.2	32.0 ± 6.2	32.0 ± 7.0	30.5 ± 10.9	27.9 ± 12.6	26.8 ± 10.3	0.38
DBLG1	32.4 ± 7.1	29.9 ± 8.8	28.8 ± 8.3	30.1 ± 9.2	30.2 ± 10.6	30.8 ± 9.7	29.5 ± 13.6	25.0 ± 9.8	0.75
P	0.0507	0.29	0.38	0.04*	0.01*	0.01°*	0.19	0.66	

Statistically significant difference between Minimed 780G and Contro-IQ: *, between Minimed 780G and DBLG1: °, between Control-IQ and DBLG1: #

The CV of CGM-measured glucose concentrations was lower, close to statistical significance (p = 0.0507), in the Minimed 780G group (29 ± 6.7%) compared to Control-IQ group (34.5 ± 6.6%) and DBLG1 group (32.4 ± 7.1%).

The other CGM metrics did not differ between the three groups (Table 2).

The insulin requirement did not significantly differ between the three groups, except between 5 and 6 am when it was greater in Diabeloop group (Table 3).

**Table 3:** Nocturnal changes in insulin infusion in three groups using different AHCL systems

AHCL system	00.00–01.00	01.00–02.00	02.00–03.00	03.00–04.00	04.00–05.00	05.00–06.00	06.00–07.00	Total
Minimed 780G group	1.6610396	1.5079352	1.2948825	1.2670534	1.1638042	1.0808221	1.4936736	9.469210
Control-IQ group	1.815943	1.6032033	1.5188858	1.2520551	1.1543655	1.1777852	1.7166627	10.238901
DBLG1 group	1.3152948	1.1398489	1.3936266	1.0445198	1.2562376	1.6514435	1.6602255	9.4611967
p value*	0.95	0.73	0.38	0.88	0.37	0.03	0.32	0.31

*AHCL* advanced hybrid closed-loop system

*Assessed using Kruskal–Wallis test

A regression analysis was performed to explore the nocturnal variation in insulin dosage across different hours of the night in all 55 patients, utilizing a panel data model (Table 4A).

**Table 4:** Nocturnal changes in insulin infusion in all participants, in DBLG1 group, in Minimed 780G group and in Control-IQ group

A. Nocturnal changes in insulin infusion in all patients—mixed-effects model			
Hours	Coefficient	95% conf. interval	p value
01.00–02.00	− 0.178	− 0.3666 to − 0.009	0.062
02.00–03.00	− 0.222	− 0.410 to − 0.034	0.020
03.00–04.00	− 0.415	−0.603 to − 0.228	0.000
04.00–05.00	− 0.431	−0.619 to − 0.243	0.000
05.00–06.00	− 0.349	−0.537 to − 0.161	0.000
06.00–07.00	− 0.053	−0.193 to − 0.182	0.955
Likelihood ratio test p value = 0.000			
Parameter test p value = 0.000			

B. Nocturnal changes in insulin infusion comparing the three AHCL systems								
AHCL system	00.00–01.00	01.00–02.00	02.00–03.00	03.00–04.00	04.00–05.00	05.00–06.00	06.00–07.00	Total
DBLG1 group	1.3152948	1.1398489	1.3936266	1.0445198	1.2562376	1.6514435	1.6602255	9.4611967
Minimed 780G group	1.6610396	1.5079352	1.2948825	1.2670534	1.1638042	1.0808221	1.4936736	9.469210
Control-IQ group	1.815943	1.6032033	1.5188858	1.2520551	1.1543655	1.1777852	1.7166627	10.238901
p value*	0.95	0.73	0.38	0.88	0.37	0.03	0.32	0.31

*Kruskal–Wallis

The mixed-effects model revealed significant variability in insulin dosage associated with different hours of the night (Likelihood ratio test p < 0.001). The estimated coefficients for each hour represent the change in insulin dosage compared to the reference hour (00.00–01.00).

**Fig. 2:**
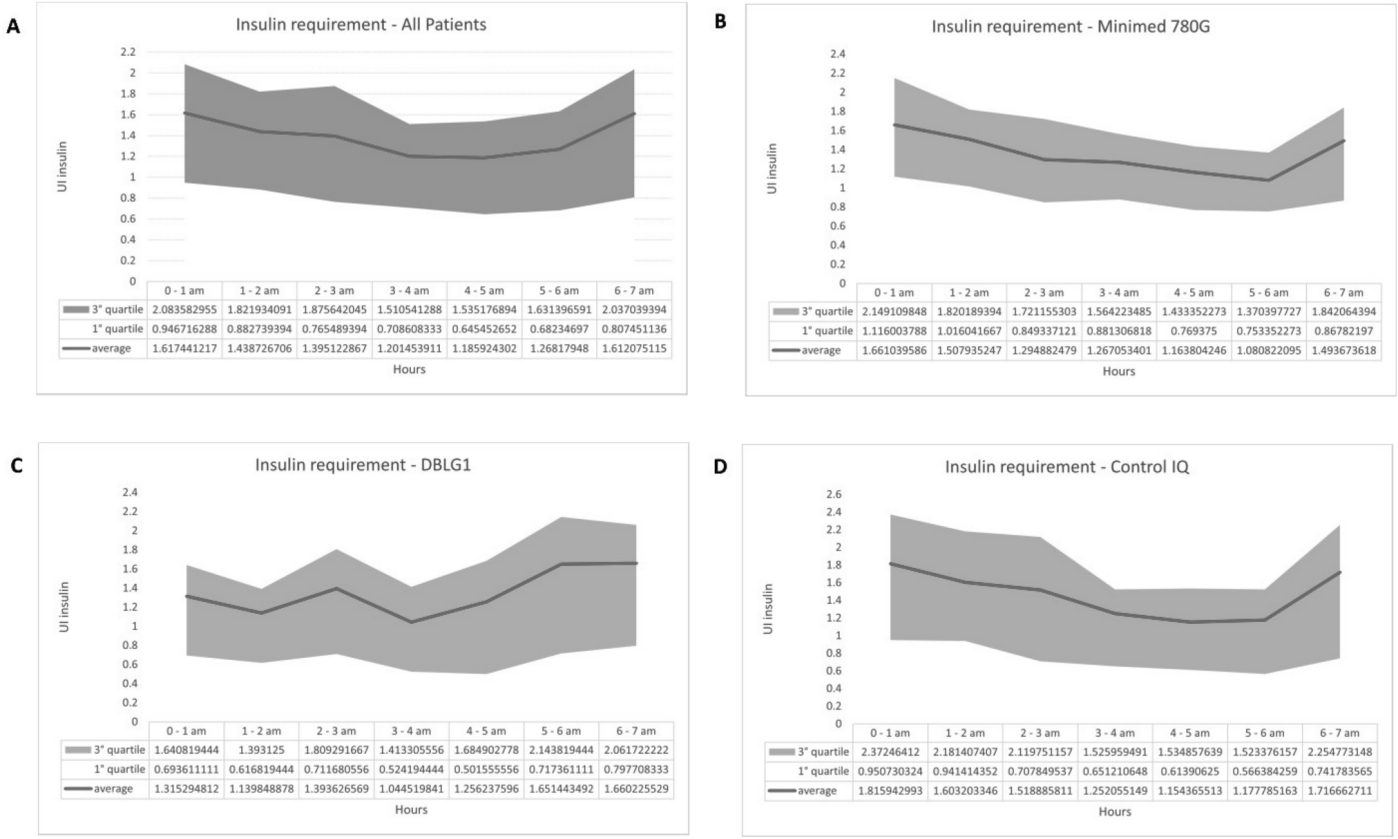
Trend of insulin administration throughout the night in all participants, in Minimed 780G group, in DBLG1 group and incontrol-IQ group

These data suggest that the time of night significantly influences insulin dosage (Fig. 2A illustrate the trend of insulin administration throughout the night in all patients). The significant negative coefficients associated with hours 02.00 to 06.00 suggest that this time interval is linked to a decrease in the insulin dosage compared to the 00.00–01.00-time interval.

Comparing the total nighttime insulin dosage administered by the three systems, there are no significant differences. However, upon analyzing specific hours of the night (Table 4), it is noteworthy that DBLG1 group exhibits a significantly higher insulin dosage administered between 05:00 and 06:00 compared to the other systems (p = 0.03), specifically 52% higher than Medtronic and 41% higher than Tandem (Fig. 2 in B, C and D shows the trend of insulin administration throughout the night across the three aHCL systems).

The hourly coefficient of variation (CV) of exogenous insulin delivery during night-time was calculated to quantity intraperson variability of insulin requirements in all participants, and in the 3 groups. The mean of intraperson variability of insulin requirement was high and similar in all hours of night-time period without difference between the three groups (Table 4).

## Discussion

To our knowledge this is the first study comparing the efficacy of three AHCL systems in optimizing glycemic control over the night-time period.

Minimed 780G group used a PID algorithm unlike group Control-IQ and DBLG1 that used a MPC algorithm.

Recently Kang et al. [11] performed a meta-analysis of randomized controlled trials of three different algorithms (MPC, PID, and Fuzzy) compared to conventional insulin therapy. The MPC algorithm-based artificial pancreas system was associated with a higher increase in TIR ([MD], 12.57%, 95% CI [9.63, 15.50] p < 0.00001) than the PID algorithm-based artificial pancreas system ([MD], 9.59%, 95% CI [− 3.67, 22.85] p < 0.00001). On the other hand, the reduction in TBR was higher in studies using the PID ([MD], − 5.24%, 95% CI [− 16.06, 5.58] p < 0.00001) algorithm than in studies using the MPC ([MD], − 1.12%, 95% CI [− 1.50 to  − 0.75] p < 0.00001) algorithm.

In a recent study Bassi et al. [9] retrospectively compared 1-year use of Tandem Control-IQ vs Minimed 780G in 76 adult and pediatric subjects with T1DM. The use of both AHCL systems led to a significant and rapid improvement of glycemic control lasting for up 12 months. Unlike in Kang’s meta-analysis, subjects using Minimed 780G with a PID algorithm obtained a higher TIR (71% vs 68%; p = 0.001) and lower TAR > 250 mg/dl (4.5% vs 9%, p = 0.009), average sensor glucose (148.5 vs 162 mg/dl, p < 0.001), and standard deviation (50 vs 58 mg/dl, p = 0.031) than users Tandem Control-IQ system with a MPG algorithm. Similarly, a prospective multicenter study, including 150 adolescents and adults with type 1 diabetes found that two AHCL (Medtronic 780G and Tandem Control-IQ) were able to provide a similar improvement in glucose control with no superiority of one system over the other [12].

However, to compare properly multiple AHCL systems it is necessary to reproduce similar conditions. As such, we evaluated adult individuals with a similar basal glucose control (HbA1c 53–58 mmol/mol or 7–7.5%): the mean GMI during the study was 55 mmol/mol (7.2%) in all three groups. The overall CGM metrics and the insulin requirement did not differ in the three group regardless of type of system and algorithm used.

We chose as main outcomes the overnight differences in CGM metrics between the three AHCL systems, because night-time data are more reproducible as there is fewer confounding external factors, such as food intake and physical activity.

In a randomized, crossover trial study Pinsker and coll. compared the efficacy of a PID vs a MPC algorithm in overnight glucose control after a standard 65 g-dinner in 20 adults with T1DM. Each participant performed two supervised 27.5 h sessions using a PID and a MPC algorithm. Overnight glucose control was excellent with both algorithms, showing similar CGM metrics [13].

Moreover, in our study the overnight glucose control was adequate and the recommended CGM targets were obtained with all three AHCL systems. The Control-IQ group showed a significantly higher percentage of time spent in tight time in range compared to other two groups. It is notable that Control-IQ technology has a “Sleep Activity Mode” to optimize glycemic control overnight. If the user activates this mode the algorithm uses a glucose target range of 112.5–120 mg/dL instead of 112.5–160 mg/dL and increases the basal insulin infusion when the predicted glucose value is > 120 mg/dL [14].

In another retrospective study the use of the Sleep Activity Mode did not lead to an increase in TIR in a pediatric population treated with Tandem–Control-IQ. However, unlike in our study the Authors did not evaluate tight TIR [15].

Sensor glucose values and insulin requirement followed a similar trend in all three groups, both decreased from midnight to 5 am. After 5 am sensor glucose values were unchanged, but insulin infusion increased displaying a reduction in insulin sensitivity in the period 4–8 a.m. as compared to the period after midnight to 3 a.m. in people with T1DM. Pioneering studies showed a reduction in insulin sensitivity in the period 4–8 a.m. in people with T1DM [16, 17].

Across the entire cohort of patients, a discernible trend indicates a decrease in insulin requirements from midnight to 6 a.m., with a more pronounced decline observed between 02:00 and 06:00. Beyond 6 a.m., there is a subsequent increase in insulin requirements.

Similarly, in non-diabetic patients, insulin secretion tends to decrease during sleep. Specifically, in the first half of the night, a reduction in insulin production may be observed as the body adapts to the diminished need for insulin in the absence of food intake. Subsequently, towards the early morning hours, endogenous insulin production may gradually increase in anticipation of the expected metabolic activity upon waking.

AHCL systems, by mimicking the physiological production of insulin, allow for optimal management of nocturnal glycemia.

A retrospective study analyzed overnight, daytime, and total insulin requirement in 32 adults with T1DM showing that overnight insulin requirements were significantly more variable (CV 31%) than daytime amounts (CV 22%) [18].

In our study the observation of an interindividual coefficient of hourly insulin requirement greater than 50% in 14 nights of observation confirms the mechanism that leads to the superiority of the aHCL system compared to traditional MDI therapy and explains the frequent difficulty of people with type diabetes 1 to obtain good glycemic control without hypoglycemia during the nocturnal period.

The main limitations of our real-life study are the retrospective design and the low number of participants. A randomized prospective study is needed to confirm our data.

In conclusion, all the three AHCL systems were equally able to achieve the recommended targets for CGM metrics overnight in adults with T1DM. The Tandem Control-IQ system obtained a higher tight TIR.

## Data Availability

All data sets generated during and/or analyzed during the present study are available from the corresponding author on reasonable request.
